# Determining Particle Size and Position in a Coplanar Electrode Setup Using Measured Opacity for Microfluidic Cytometry †

**DOI:** 10.3390/bios11100353

**Published:** 2021-09-23

**Authors:** Douwe S. de Bruijn, Koen F. A. Jorissen, Wouter Olthuis, Albert van den Berg

**Affiliations:** BIOS Lab-on-a-Chip Group, MESA+ Institute for Nanotechnology, Max Planck Center for Complex Fluid Dynamics, University of Twente, P.O. Box 217, 7500 AE Enschede, The Netherlands; k.f.a.jorissen@utwente.nl (K.F.A.J.); w.olthuis@utwente.nl (W.O.); a.vandenberg@utwente.nl (A.v.d.B.)

**Keywords:** impedance cytometry, microfluidics, coplanar electrodes, electrical opacity, particle tracking, positional dependence

## Abstract

Microfluidic impedance flow cytometers enable high-throughput, non-invasive, and label-free detection of single-cells. Cytometers with coplanar electrodes are easy and cheap to fabricate, but are sensitive to positional differences of passing particles, owing to the inhomogeneous electric field. We present a novel particle height compensation method, which employs the dependence of measured electrical opacity on particle height. The measured electrical opacity correlates with the particle height as a result of the constant electrical double layer series capacitance of the electrodes. As an alternative to existing compensation methods, we use only two coplanar electrodes and multi-frequency analysis to determine the particle size of a mixture of 5, 6, and 7 µm polystyrene beads with an accuracy (CV) of 5.8%, 4.0%, and 2.9%, respectively. Additionally, we can predict the bead height with an accuracy of 1.5 µm (8% of channel height) using the measured opacity and we demonstrate its application in flow cytometry with yeast. The use of only two electrodes is of special interest for simplified, easy-to-use chips with a minimum amount of instrumentation and of limited size.

## 1. Introduction

Cell separation and sorting are essential procedures in cell biology, cellular therapies, and diagnostics. In these fields, analysis of the heterogeneity of the studied sample is of special interest [1,2,3]. Traditional methods like fluorescence activated cell sorters (FACSs) or magnetic activated cell sorters (MACSs) are powerful tools to detect cell phenotypes, but they need cell-labelling, are complex, and are not cost-effective. Fortunately, microfluidic systems and microscale technology have the potential of increasing automation and reducing costs [2,4,5]. In this work, we focus on electrical impedance flow cytometry. Electrical impedance flow cytometry offers non-invasive and label-free analysis of single cells in terms of size and dielectric properties, which enables a wide variety of biological cell studies [6,7,8,9], but loses some sensitivity and specificity compared with labeling techniques. In general, two types of electrode configurations can be distinguished within electrical flow cytometers: planar (facing each other) or coplanar (side by side). Planar electrodes have a higher sensitivity, but a complex fabrication process, whereas coplanar electrodes are less sensitive, but simpler and cheaper to fabricate [10,11]. In both configurations, the position of particles is an important parameter for an accurate analysis of these passing particles or cells, as recently discussed by Daguerre et al. [12].

**Figure 1:**
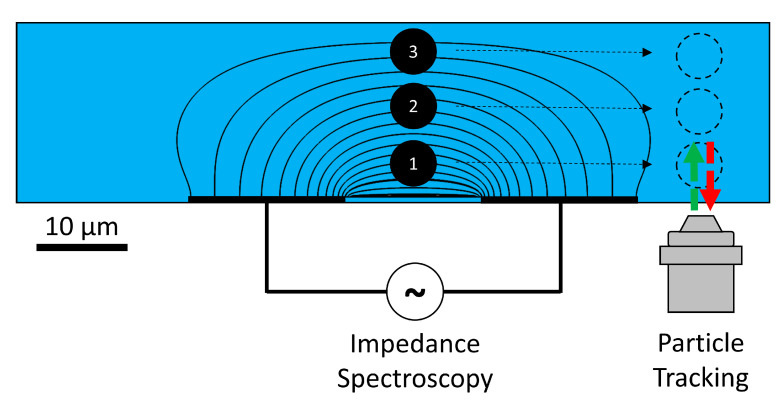
Coplanar electrode setup illustrating the inhomogeneous electric field. Passing particles in laminar flow at the bottom, middle, and top of the channel will all result in different impedance changes, owing to the inhomogeneity. Independent optical verification of the particle height is performed using general defocusing particle tracking (GDPT).

Impedance measurements are particularly sensitive to positional differences in regions where the electric field is inhomogeneous, which is common for coplanar electrode setups (see Figure 1). Gawad et al. [10] showed with finite element method (FEM) simulations that the impedance response at high frequency over the impedance response at low frequency (known as opacity [13]) was barely influenced by the particle position and largely independent of the particle size, although ignoring the effect of the electrical double layer (EDL). Recent findings stress the importance of the EDL’s capacitive behavior for systems with an inhomogeneous electric field; a strong correlation between the measured opacity and particle position was found [14]. It is important to note that the opacity, a material property, of a particle cannot be measured independently of the measurement setup; therefore, we refer to this as the measured opacity [14]. This phenomenon could explain unexpected large spreads in the opacity of polystyrene beads when one does not compensate for the position (e.g., Kirkegaard et al. [15]).

One way to overcome the positional variability is by particle focusing [5,16] like hydrodynamic sheath flow focusing [17,18], inertial focusing [19,20], or dielectrophoretic focusing [21,22]. Unfortunately, this introduces additional fluid channels or electrodes and, thereby, extra complexity.

Fortunately, the correlation between measured opacity and particle position enables a new compensation strategy to account for the particle position in a focus-free system, similar to existing work by, e.g., the group of Caselli [23,24,25,26]. These previous methods exploit the signal shape to find a metric that correlates with the particle trajectory. The reported methods are successful and show particle diameters close or equal to the manufacturer’s specifications using at least three electrodes. Using our opacity-based alternative compensation strategy, we can adjust for the inhomogeneous electric field and determine the particle size in an easy to fabricate coplanar setup with only two electrodes.

In addition to our previously published paper [14], we compensate the electrical diameter of polystyrene beads and yeast for their vertical position, and we independently verify and quantify the relation between the measured opacity and the particle height using general defocusing particle tracking (GDPT [27]). Furthermore, we present an equivalent circuit model to extend upon the previously used FEM simulation. In this paper, we first report the use of measured opacity as a successful compensation strategy to account for particle position using only two coplanar electrodes and multi-frequency analysis. Additionally, we propose the use of the measured opacity as a novel and reliable method to determine the vertical position of passing beads. This will be investigated by simultaneously performing multi-frequency impedance measurements to measure the electrical opacity and GDPT as an independent optical verification method for the particle height. Finally, a verification experiment with yeast cells [24,25,28] is performed to show its possible application in flow cytometry.

## 2. Theory

In coplanar electrode setups, monodisperse particles display a wide spread in measured diameter, owing to different positions in the inhomogeneous electric field between the electrodes, as illustrated in Figure 1. To find a constant diameter for monodisperse beads of a certain size, we deploy a compensation method based on the correlation between measured opacity and particle height [29]. Electrical opacity, *O*, can be defined as the change in impedance magnitude Δ|*Z*| at high over low frequency [13]:

(1) $$O=\frac{\Delta \left|Z\right|\left(\mathrm{high}\;\mathrm{freq}\right)}{\Delta \left|Z\right|\left(\mathrm{low}\;\mathrm{freq}\right)}.$$

The correlation between measured opacity and particle height is the result of the interaction between the change in resistance and the capacitive double layer, as we discuss in more detail using an equivalent circuit model (ECM).

### 2.1. Equivalent Circuit Model

**Figure 2:**
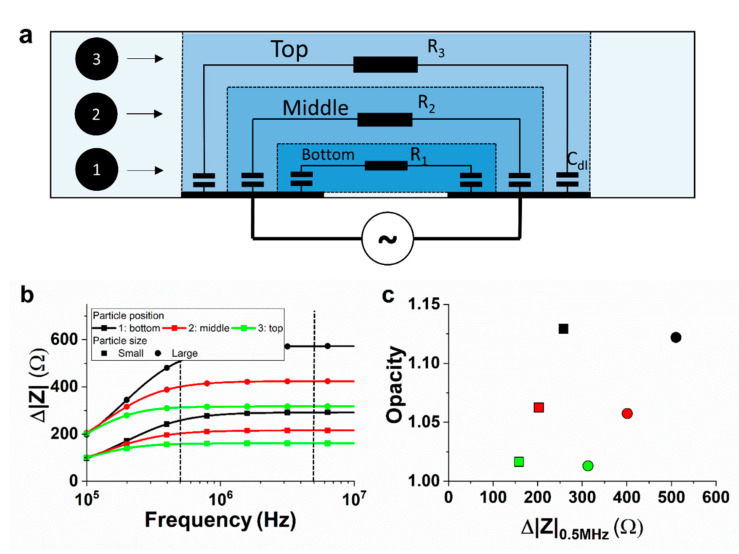
(**a**) Illustration of the ECM. The system was modelled using three parallel RC networks to model three different positions of passing particles. (**b**) Impedance response of the two sizes of particles at different positions. (**c**) Opacity versus the impedance change for the different positions. Opacity is defined as the ratio of the absolute impedance change at 5 MHz over 0.5 MHz (black dotted lines in (**b**)). Parameters used: *C*_dl_ = 20 pF, *R*_1_ = 72 kΩ, *R*_2_ = 80 kΩ, and *R*_3_ = 88 kΩ, and the changes in resistance Δ*R_n_* at the bottom, middle and top position are 2200, 2000, and 1800 Ω, respectively, for the small particle and 4400, 4000, and 3600 Ω, respectively, for the large particle. The top path length is considered to be 10% longer than the middle path and the bottom path 10% shorter than the middle path.

The importance of including the electrical double layer to understand the measured opacity of particles in coplanar electrode setups has been demonstrated with FEM simulations in earlier work [14]. Here, we illustrate our understanding using a basic ECM to describe the frequency response of a three-layered RC network shown in Figure 2a, representing a simplified setup with three fluidic layers. These models have previously been used to describe the frequency response of systems with electrical field gradients [30,31].

Each fluidic layer is modelled by a double-layer capacitance *C*_dl_ in series with a medium resistance *R_n_* (for layer *n*). The double-layer capacitance ranges from 0.1 to 0.4 F/m^2^ depending on, among other things, the applied potential and ionic concentration [32]. In our case, the double-layer behaviour is approached by a capacitor of constant value $C_{\mathrm{dl}}=C_{\mathrm{sp}}A$, where *C*_sp_ is the specific capacitance and *A* the area of the electrode/electrolyte interface. The exposed area is equal for all three layers, thus *C*_dl_ is constant for each layer. The three layers do have different medium resistances, because of different path lengths. The layers can be modelled as resistive elements $R_{n}=\frac{1}{\sigma }\frac{l_{n}}{A}$ with *σ* being the medium electrical conductivity (S/m), *l_n_* the path length, and *A* the area of the electrode/electrolyte interface. The exposed area *A* is again taken equal for all three layers. The bottom layer (*n* = 1) has the shortest path length and thus the least resistance. The top layer (*n* = 3) has the longest path length and thus the most resistance. The total impedance of the system without a particle, *Z*_0_, can be described by the following parallel RC network:

(2) $$Z_{0}=\left(R_{1}+Z_{\mathrm{dl}}\right)//\left(R_{2}+Z_{\mathrm{dl}}\right)//\left(R_{3}+Z_{\mathrm{dl}}\right).$$

The last step is to include the change in impedance, because of the passage of a particle within layer *n*. The induced impedance change is dependent on the particle volume and the medium volume. For a particle of certain size, we expect the smallest impedance change in the top layer and the largest impedance change in the bottom layer, because of the difference in medium volume. This is modelled by adding a resistance Δ*R_n_* to the medium resistance *R*_n_, which scales inversely with the path length *l_n_* of the specific layer. For example, when the passage of a particle at the bottom layer is modelled:

(3) $$Z_{with\;particle}\left(n=1\right)=\left(R_{1}+\Delta R_{1}+Z_{\mathrm{dl}}\right)//\left(R_{2}+Z_{\mathrm{dl}}\right)//\left(R_{3}+Z_{\mathrm{dl}}\right).$$

The absolute change in impedance Δ|*Z*|(*n*) is now expressed as the difference between the total impedance with particle *Z*_with particle_ (*n*) and without *Z*_0_:

(4) $$\Delta \left|Z\right|\left(n\right)=Z_{\mathrm{with}\;\mathrm{particle}}\left(n\right)-Z_{0}.$$

The calculated impedance response of a small- and large-sized particle is shown in Figure 2b for all three particle positions. The maximum impedance change scales with the particle size and the relative position to the electrodes, as expected. Furthermore, the impedance response at a low frequency is dominated by the electrical double layer, but the cut-off frequency changes notably for different particle positions. This results in different opacities, defined here as Δ|*Z*|_5MHz_/Δ|*Z*|_0.5MHz_ and indicated by the vertical dotted lines in Figure 2b. On the contrary, the particle size does not influence the frequency response notably and, with that, neither the opacity. The resulting opacity with respect to the impedance change at 0.5 MHz is given in Figure 2c for the different particle sizes and positions. The correlation between the opacity and position is similar to the correlation previously found in the literature using FEM simulations [14].

It becomes apparent that we can directly differentiate between the “small” and “large” particle despite the variations in particle position, because the opacity gives information about the position, whereafter the impedance change reveals the particle size. Next, we are going to use these properties to find the actual size of passing particles.

### 2.2. Opacity Compensation

The electrical diameter *D* of a passing spherical particle is estimated as the cubic root of the absolute impedance change Δ|*Z*|, as shown in Equation (5) [24,25]:

(5) $$D=G\Delta {\left|Z\right|}^{\frac{1}{3}},$$

where *D* is the electrical diameter in µm, *G* is the gain factor in µm/Ω^1/3^, and Δ|*Z*| is the absolute impedance change in Ω. The gain factor compensates for the electronic circuitry, channel geometry, and medium properties. A linear relationship between the measured opacity *O* and the normalized electrical diameter *D*/*d* is assumed to correct for the particle position:

(6) $$O=c_{1}D_{\mathrm{measured}}/d+c_{2},$$

where *d* is the nominal size of the beads in µm and fitting parameters *c*_1_ and *c*_2_. Finally, the corrected electrical diameter *D*_corrected_ can be expressed as follows:

(7) $$D_{\mathrm{corrected}}=\frac{c_{1}D_{\mathrm{measured}}}{O-c_{2}}.$$

We continue with an experimental validation of the proposed compensation strategy.

## 3. Materials and Methods

### 3.1. Device Fabrication

**Figure 3:**
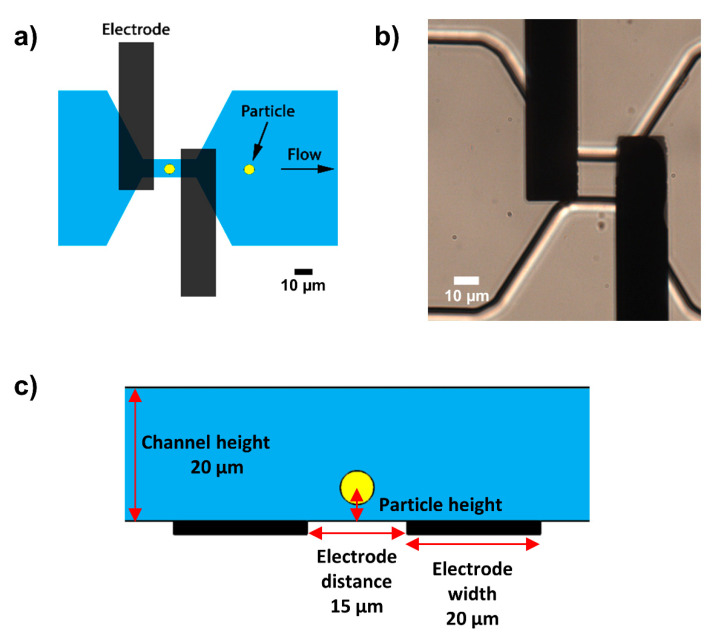
(**a**) Top view of the microfluidic chip showing the coplanar electrode pair and constriction channel to increase the sensitivity of the measurement. (**b**) Realization of the glass-PDMS chip with coplanar electrodes. (**c**) Side view of the chip with a channel height of 20 µm, electrode distance of 15 µm, and the particle height relative to the electrodes.

A simple coplanar electrode setup was used as described previously [14,33]. The glass-PDMS chip consists of a constriction channel and two tantalum/platinum electrodes, as illustrated in Figure 3.

### 3.2. Sample Preparation

Previous experiments with this device were performed using seawater to study algae [14]. For this reason, we started using the same protocols in our experiments with beads, including the use of seawater. The conductivity of seawater is 4 S/m, comparable to other conductive electrolytes like phosphate buffered saline (PBS) solutions, which is often used in other studies (e.g., [23,34]) and which we will later use in our experiment with yeast cells.

Polystyrene beads of 5, 6, and 7 µm (Sigma-Aldrich, Buchs, Switzerland and PolySciences, Warrington, PA, USA) were diluted in seawater to a density of approximately 5 × 10^6^ beads/mL. Clumping of beads was prevented by adding 0.1% Tween 20 to the sample solution and by performing 5 min of sonification of the sample solution before the experiment. For particle tracking, red fluorescent (Ex/Em 530/607 nm) polystyrene beads of 5 µm (Microparticles GmbH, Berlin, Germany) were treated in the same way, but used at a lower concentration (~1 × 10^6^ beads/mL) to ensure correct assignment of each tracked particle to the corresponding impedance response. The mass density of the polystyrene beads is 1.05 g/cm^3^, which is comparable to the mass density of seawater (1.03 g/cm^3^).

Fresh baker’s yeast (*Saccharomyces cerevisiae*) was bought at a local grocery store and diluted in PBS solution (Sigma-Aldrich) to a density of approximately 1 × 10^6^ cells/mL. The typical diameter of Baker’s yeast cells is 5–10 µm for large cells and 1–7 µm for small cells [35]. The sample was spiked with 5 µm polystyrene beads (diluted in PBS and containing 0.1% Tween 20). The mass density of the solution was increased using sucrose to approach the mass density of the polystyrene beads and yeast cells (~1.1 g/cm^3^ [36]). The conductivity of the solution was 1.2 S/m (conductivity probe LE703, Mettler Toledo, Greifensee, Switzerland). Visual inspection showed little to no budding cells. The samples were immediately introduced into the measurement setup after the described preparation steps.

The microfluidic chip was treated with a monolayer surface coating (0.1 mg/mL PLL-g-PEG, SuSoS, Dübendorf, Switzerland) before the experiment to prevent the beads from sticking to the channel. A constant flow of 0.05 µL/min was initiated throughout the experiments with a neMESYS syringe pump (Cetoni, Korbussen, Germany).

### 3.3. Data Acquisition and Processing

Impedance data were recorded using a HF2LI lock-in amplifier (Zurich Instruments, Zurich, Switzerland) and a HF2TA transimpedance amplifier (Zurich Instruments). The lock-in amplifier allows for simultaneous recording of multiple signal frequencies. Here, 0.5 and 20 MHz signals were applied during the optical particle tracking experiment, while the other experiments with beads were performed at 0.5, 1, 4, 12, and 20 MHz. The signal amplitude was set to 1 V peak to peak and the sample rate was 28.8 kSa/s for all experiments.

The impedance data were post processed in MATLAB (R2020a, MathWorks, Natick, MA, USA) to determine the peak height Δ|*Z*| of passing beads. Determination of the peak height of particles passing close to the electrodes requires extra attention, because of the M-shaped response (Figure S1) [37]. A more extensive explanation of the post processing method can be found in the Supplementary Information.

The fitting parameters *c*_1_ and *c*_2_ in Equation (6) were determined with a robust fit on the measured normalized electrical diameter. The performance of the compensation strategy was assessed by calculating the standard deviations of the particle distributions using a Gaussian fit in Origin (2019b, OriginLab Corporation, Northampton, MA, USA).

The GDPT procedure is described by Barnkob et al. [27] and is performed using fluorescent imaging. The fluorescent beads were imaged using a Leica DMi 5000M microscope (Amsterdam, the Netherlands) at 63X magnification (dry lens), a CoolLed pE-300^Ultra^ light source (Andover, UK), a Leica N2.1 filtercube (Amsterdam, the Netherlands), and a Hamamatsu ORCA-Flash4.0 digital CMOS camera (Hamamatsu-city, Japan). An exposure time of 1 ms was used at 142 FPS. The calibration series consisted of 16 images at 1 µm spacing of a single immobilized 5 µm fluorescent particle on the glass bottom of the filled fluidic channel.

The GDPT output was post processed in MATLAB. Each particle was tracked in multiple frames as they move through the channel. To prevent accidental double registrations, false registrations, and other anomalies, a minimum of eight consecutive particle observations was set for the registration of a single particle to be further processed. The particle height was taken as the average height of all these observations. Finally, the particle height was matched to the measured electrical opacity of a particle that was closest in time. Origin software was used to perform linear regression analysis on the relationship between the particle height and measured opacity.

The experiments with yeast cells were performed at 120 kHz, 750 kHz, and 20 MHz. The opacity compensation was performed using the 120 kHz (based on approximately half the cut-off frequency determined by the medium resistance in series with the electrical double layer capacitance) and 750 kHz signal (below the cell’s β-dispersion, <several MHz [38]), whereas the 20 MHz signal was needed to differentiate between the beads and yeast cells.

## 4. Results and Discussion

A typical impedance–time response, including the peak detection, of a bead at low and high position in the channel can be found in the Supplementary Information. Here, we continue with the discussion of the detected beads. First, we focus on the compensation method, after which we verify and quantify the measured opacity–position relationship, as published previously [14] using GDPT. Finally, we show the applicability with yeast experiment.

**Figure 4:**
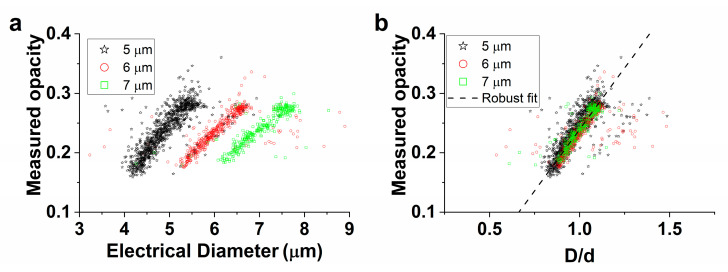
(**a**) Measured opacity as a function of the electrical diameter of 5, 6, and 7 µm monodisperse beads. (**b**) Measured opacity versus the normalized electrical diameter D/d for the same populations of beads as in (**a**). A robust fit was used to determine the fitting parameters *c*_1_ and *c*_2_ in Equation (6) for each set of beads. The displayed fit is the average of these three sets. The electrical diameter was based on the 0.5 MHz impedance signal and the opacity was expressed as Δ|*Z*|_20MHz_/Δ|*Z*|_0.5MHz_.

The measured opacity (Δ|*Z*|_20MHz_/Δ|*Z*|_0.5MHz_) versus the (normalized) electrical diameter of 5, 6, and 7 µm beads is displayed in Figure 4a,b. What can clearly be seen in Figure 4a are the distinct clusters for all sets of beads, as expected from theory (Figure 2c). The separate measurements of 5, 6, and 7 µm beads in Figure 4b were used to fit the parameters *c*_1_ and *c*_2_ (see Equation (6)). The result of the robust fit for each measurement set and the average of these results (‘all’) are shown in Table 1.

**Figure 5:**
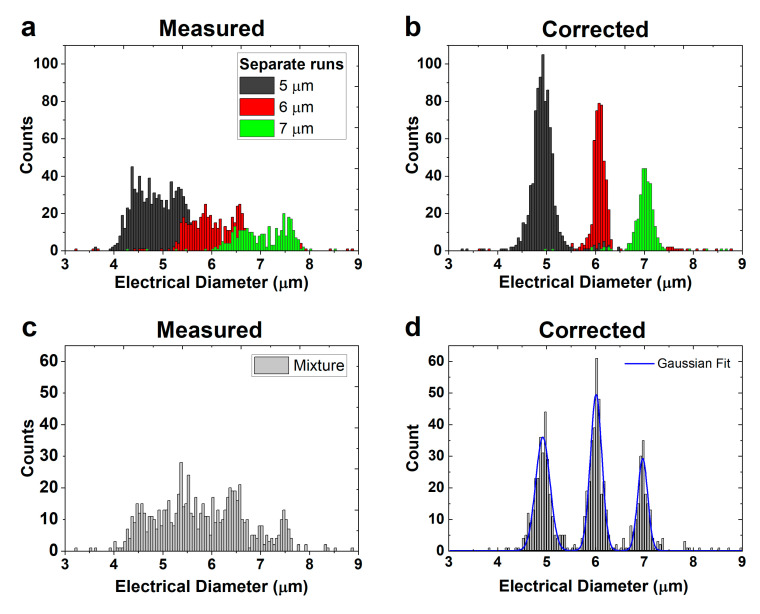
(**a**) Spread in electrical diameter of three separate runs of 5, 6, and 7 µm beads. (**b**) The corrected electrical diameter of the beads in (**a**). (**c**) Spread in electrical diameter of a mixture of 5, 6, and 7 µm beads. (**d**) The corrected electrical diameter of the mixture of beads with a Gaussian fit.

After determining the average fitting parameters (‘all’), the opacity compensation (Equation (7)) was performed. The results before (measured) and after (corrected) opacity compensation of the measurements in Figure 4 are shown in Figure 5a,b.

Another measurement was performed, but now with a mixture of the 5, 6, and 7 µm beads. The size distribution of >750 beads, before and after opacity compensation, is shown in Figure 5c,d, respectively. The quality of the three peaks in Figure 5d was assessed using a Gaussian fit to find the standard deviation of the peaks. The standard deviations of the 5, 6, and 7 µm beads are 0.29 µm (CV = 5.8%), 0.24 µm (CV = 4.0%), and 0.20 µm (CV = 2.9%), respectively, which is slightly larger than the manufacturer’s specifications (<3.1%, <2.8%, and <1.5%). Our method does not improve the CV compared with other compensation methods in the literature (see Table S1), but requires fewer electrodes. A literature overview is given in the Supplementary Information.

**Figure 6:**
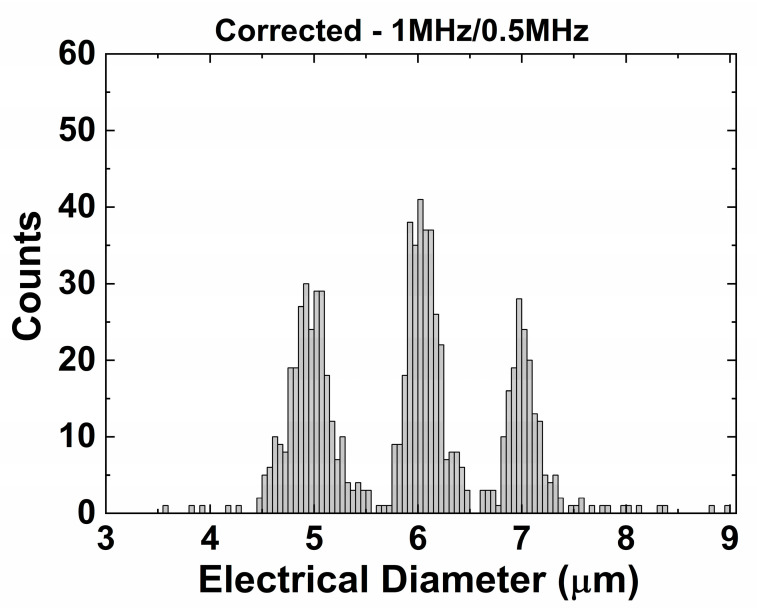
Opacity compensation using the same dataset as shown in Figure 5c. The 1 MHz signal was used as higher frequency to perform the opacity compensation.

The opacity compensation shows a comparable result for frequencies of 0.5 and 1 MHz (Figure 6). An extended frequency analysis (for 0.5, 1, 4, 12, and 20 MHz) can be found in the Supplementary Information, illustrating that the lower frequency should be below 1 MHz to maintain a good separation for this system. As a result, this compensation method can be used for particles, cells, and the mix of those, as long as we choose our frequencies below the β-dispersion of cells (<several MHz [38]), which we will illustrate later with a mixture of beads and yeast cells.

**Figure 7:**
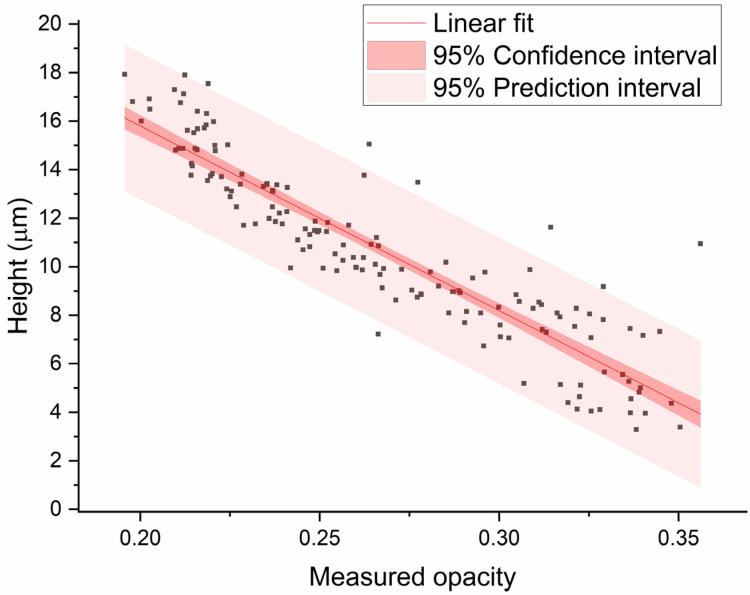
Measured bead height (relative to the electrodes) versus measured opacity (Δ|*Z*|_20MHz_/Δ|*Z*|_0.5MHz_) for 152 beads. Linear regression analysis was performed to find the 95% confidence and prediction interval. The mean standard deviation (RMSE) of the fit is 1.5 µm (8% of the channel height) and R^2^ = 0.83.

Next, the bead height was optically determined by GDPT. The bead height versus the measured opacity is plotted in Figure 7 and reveals the expected correlation: the closer the bead is to the electrode, the higher the measured opacity. Using linear regression analysis, a standard deviation (RMSE) of 1.5 µm was found, which gives an accuracy of 8% of the channel height. This is comparable to the most accurate impedance technique reported in the literature (best 8% of channel height), as shown in a short literature survey in the supplement of Reale et al. [39] However, our presented setup is not able to determine the lateral position. The 3D particle tracking method was able to determine the bead height with an average standard deviation of 0.78 µm.

Lastly, a verification experiment with yeast cells was performed [24,25,28]. First, the fitting parameters were determined by introducing 5 µm polystyrene beads into the system (see supplement). The measured opacity was calculated at a low frequency (Δ|*Z*|_750kHz_/Δ|*Z*|_120kHz_) to focus merely on variations due to the particle’s position and thus below the cell’s β-dispersion (<several MHz [38]) to prevent influences of the true opacity of yeast cells.

**Figure 8:**
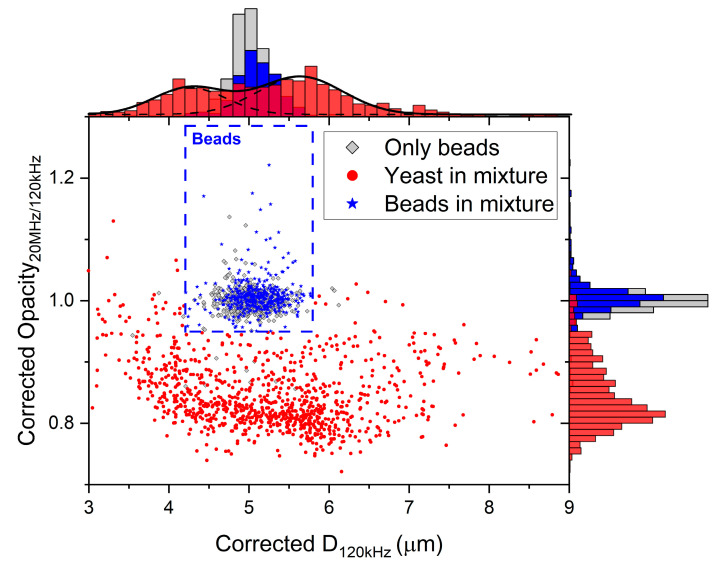
Corrected opacity (Corrected *D*_20MHz_/Corrected *D*_120kHz_) versus the corrected diameter *D* at 120 kHz. The beads in the mixture (n = 389) were differentiated from the cells (n = 947) with a threshold (corrected opacity >0.95 and 4.2 µm < corrected *D*_120kHz_ < 5.8 µm) comparable to the measurements of only beads (n = 592).

Next, a mixture of yeast cells and polystyrene beads was introduced. The measured electrical diameter was corrected at 120 kHz and 20 MHz using the fitting parameters calculated previously. Figure 8 shows a significant difference in ‘corrected opacity’ between beads and yeast cells. The ‘corrected opacity’ is defined as the corrected diameter at a high frequency (20 MHz) over the corrected diameter at a low frequency (120 kHz). The beads in the mixture were differentiated from the yeast cells based on their opacity (>0.95) and size (4.2 µm < corrected *D*_120kHz_ < 5.8 µm). The size is represented by the corrected diameter at low frequency (120 kHz). Variations within a population of yeast cells might explain the spread in corrected opacity. Furthermore, the measured yeast cells can be differentiated into two populations, which vary in size [35]. Two Gaussian distributions have been fitted accordingly (mean = 4.3 ± 0.9 µm and mean = 5.6 ± 1.1 µm, respectively).

Which conditions need to be fulfilled to use this compensation method? First of all, it requires an inhomogeneous E-field, meaning that the electrodes are close together with respect to the channel height. Secondly, the low frequency should be selected at approximately half the cut-off frequency (*f*_cut_ = 1/(*RC*)) of the EDL capacitance and the medium resistance. The selection of this frequency depends on the electrode material, electrode size, medium conductivity, and geometry. In general, an increase in EDL capacitance (large surface area or large specific capacitance) or an increase in medium resistance (medium conductivity and geometry) will cause a downward shift in this frequency. Lastly, the high frequency should be selected below the β-dispersion of the cells under study and should not be too close to the low frequency. Furthermore, the electrical properties of the particles should be different from the medium.

## 5. Conclusions

To conclude, we have implemented a new compensation strategy to determine the size of particles in the inhomogeneous electric field of a coplanar electrode setup without particle focusing, using the measured opacity. This compensation strategy requires only two simple coplanar electrodes and a multi-frequency impedance analysis. A mixture of 5, 6, and 7 µm polystyrene beads was separated with an accuracy (CV) of 5.8%, 4.0%, and 2.9%, respectively, which is close to the specifications of the manufacturer.

The observed relation between the measured opacity, impedance change, and particle position is in agreement with the proposed ECM. These observations stress the important role of the capacitive EDL at a low frequency in systems with coplanar electrodes.

Additionally, we have independently confirmed the strong correlation between bead height and measured opacity, using the optical technique of GDPT. We were able to predict the bead height with an accuracy of 1.5 µm (8% of channel height) using the measured opacity.

Finally, we have demonstrated the applicability in flow cytometry with yeast cells that, independent of their position, can easily be distinguished from 5 µm polystyrene beads. The measured size of polystyrene beads and yeast cells can be corrected for their position simultaneously, as long as the measured opacity is calculated below the β-dispersion of the cells under study.

Impedance signals are very rich and treasure valuable information about both the measurement system and the particles under study. This simple coplanar electrode setup utilizes the EDL capacitance and the inhomogeneous electric field to accurately determine the particle size and position using the measured opacity. The use of only two electrodes can be of special interest for simplified, easy to use chips with a minimum amount of instrumentation and of limited size.

## Figures and Tables

**Table 1:** Fitting parameters of the linear robust fit (*O* = *c*_1_*D*/*d* + *c*_2_) and the gain factor *G* for the separate measurements in Figure 4a,b. *G* was chosen, such that the mean measured electrical diameter *D* equals the nominal diameter *d*.

*d* (µm)	*c*_1_	*c*_2_	*G* (µm/Ω^1/3^)
5	0.40	−0.15	0.70
6	0.40	−0.17	0.68
7	0.39	−0.15	0.69
All	0.40	−0.16	0.69

## Data Availability

Data available on request.

## Supplementary Materials

The following are available online at https://www.mdpi.com/article/10.3390/bios11100353/s1, Figure S1: Impedance response bead. Figure S2: Compensation strategy frequency analysis. Figure S3: Optical investigation yeast. Figure S4: Fitting parameters yeast experiment. Figure S5: Corrected diameters yeast experiment.
