# Supplementary material for: Determining Particle Size and Position in a Coplanar Electrode Setup Using Measured Opacity for Microfluidic Cytometry
Source: Biosensors (Basel). 2021 Sep 23;11(10):353. doi: 10.3390/bios11100353 (PMC8533872; doi:10.3390/bios11100353)
Supplement: Supplementary file 1 [file biosensors-11-00353-s001.zip › biosensors-1380458-supplementary.pdf]

Supplementary Information

Determining Particle Size and Position in Coplanar Electrode Setup using Measured Opacity for Microfluidic Cytometry

Douwe S. de Bruijn[a,*], Koen F.A. Jorissen[a], Wouter Olthuis[a], Albert van den Berg[a]
[a] BIOS Lab-on-a-Chip group, MESA+ Institute for Nanotechnology, Max Planck – University of Twente Center for Complex Fluid Dynamics, University of Twente, P.O. Box 217, 7500 AE Enschede, The Netherlands,
[*]Corresponding author: d.s.debruijn@utwente.nl

Peak detection method

At first, the absolute impedance $|Z|$ was calculated, where after the baseline was removed using a 6th order polynomial fit. The resulting absolute impedance change $\Delta|Z|$ over time is shown for two passing beads in Figure S1. We identified two typical impedance responses: a single peak and a M-shaped peak. The M-shaped peak is the result of beads passing very close to the two electrodes [1], causing two peaks (I and III) and a local minimum (II). A fair comparison between the change in impedance between a single and M-shaped peak was accomplished by detecting the maximum of the single peak and the local minimum of the M-shaped peak, which coincides with approximately the same location (II) between the two electrodes (see inset Fig. S1). As a result, the opacity of beads with the M-shaped response was defined as the ratio of the two minima at 20 MHz and 0.5 MHz. Matlab's peak find algorithm was used to define the type of impedance response (number of maxima within a certain time window).

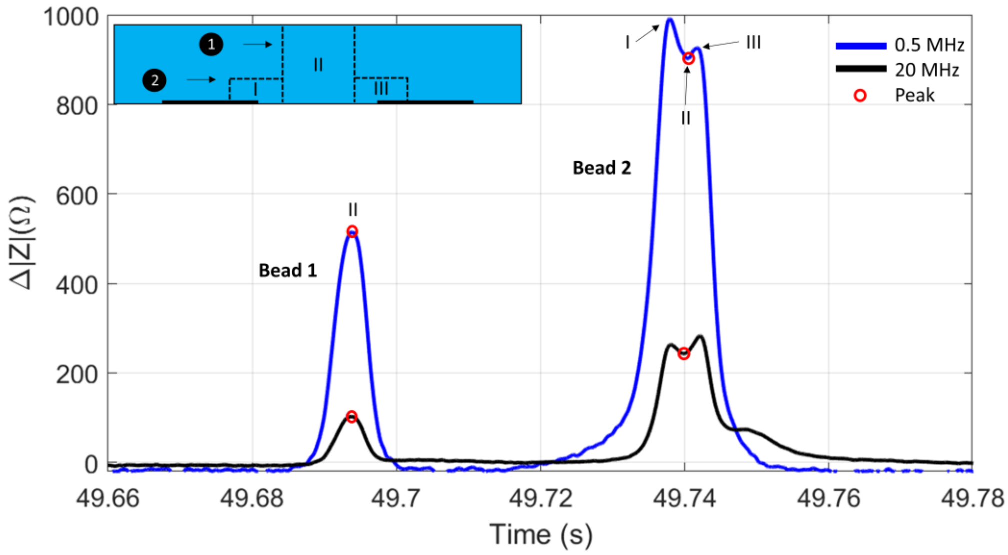

Figure S1: Typical impedance response at 0.5 and 20 MHz for two 6 µm beads passing at different heights. Two distinct responses can be identified: first a single peak and second a M-shaped peak. The first bead (opacity = 0.20) passes relatively high in the channel (far from the electrodes), whereas the second bead (opacity = 0.27) passes at the bottom of the channel (close to the electrodes). The registered absolute impedance change Δ|Z| ('Peak') for each bead and at each frequency is indicated with a red circle.

Frequency dependence of compensation strategy

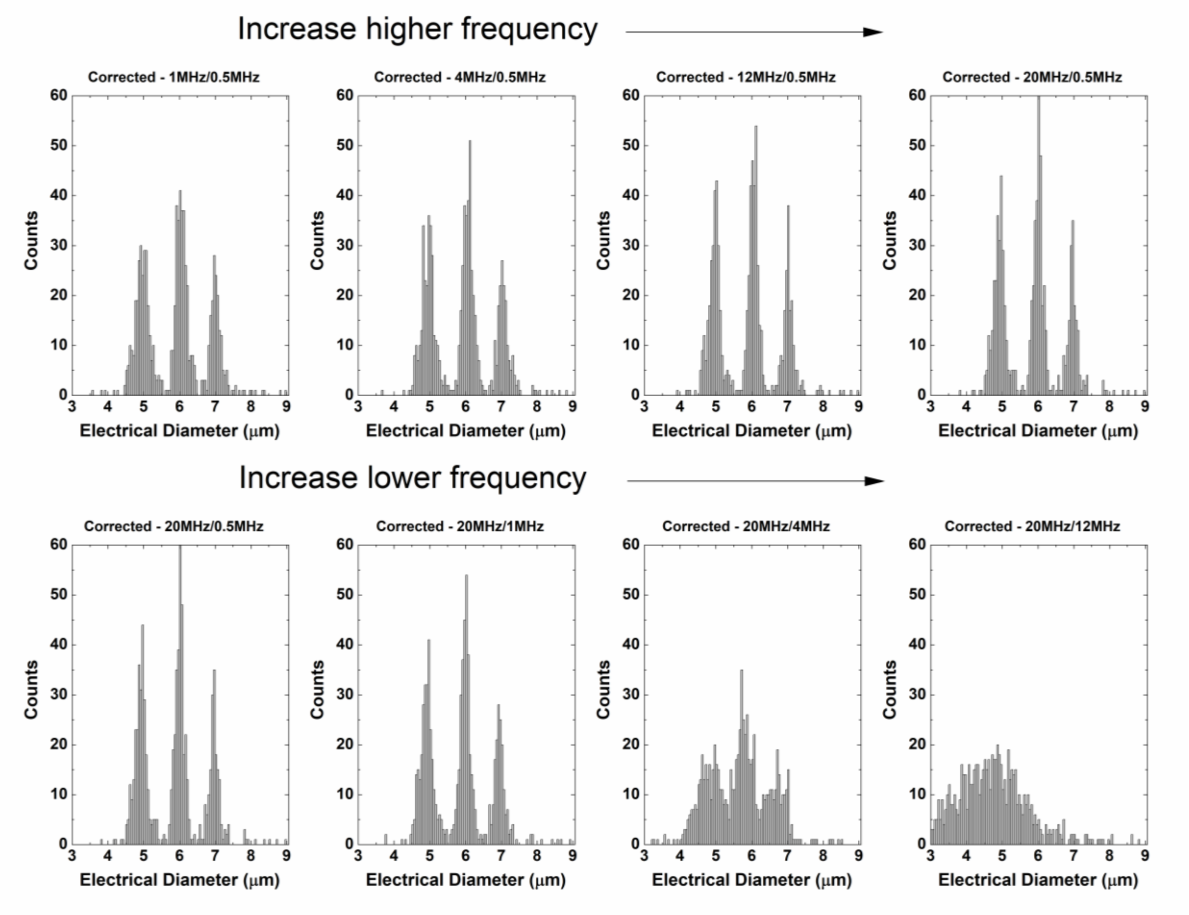

Figure S2: Compensation strategy performed at different lower and upper frequencies of the opacity. The bottom figures show a degrading quality of separation when the lower frequency increases, stressing the importance of the double layer capacitance at low frequency.

Additional figures yeast experiment

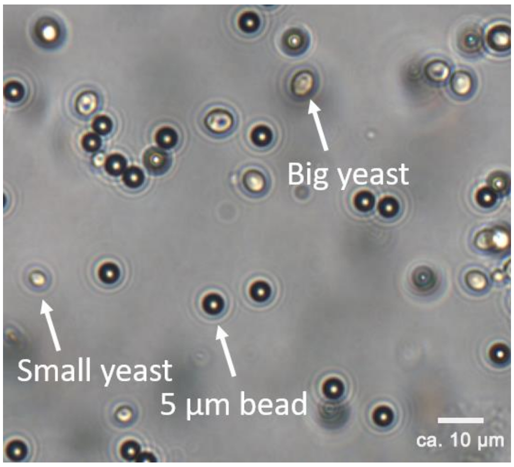

Figure S3: Sample under study, 5 μm PS beads and yeast cells diluted in PBS.

The opacity compensation was performed using the 120 and 750 kHz signal to correct the measured electrical diameter:

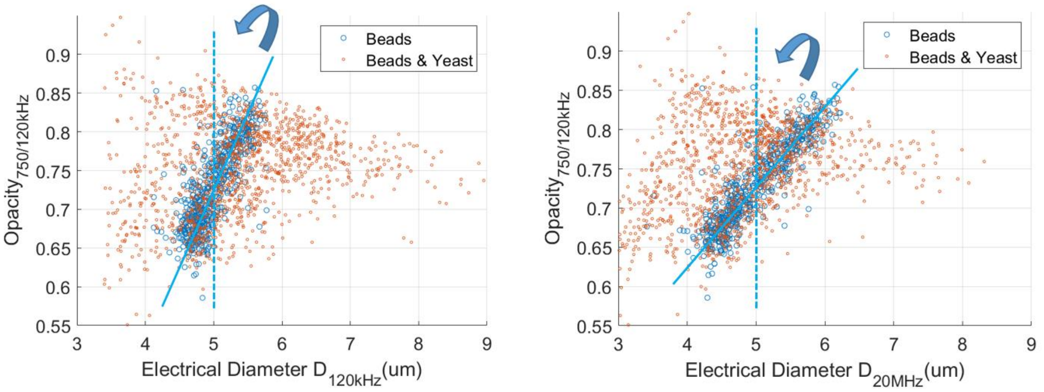

Figure S4: The fitting parameters for correction of the electrical diameter at 120 kHz (left) and 20 MHz (right) were determined using a run with only 5 µm PS beads (in blue).

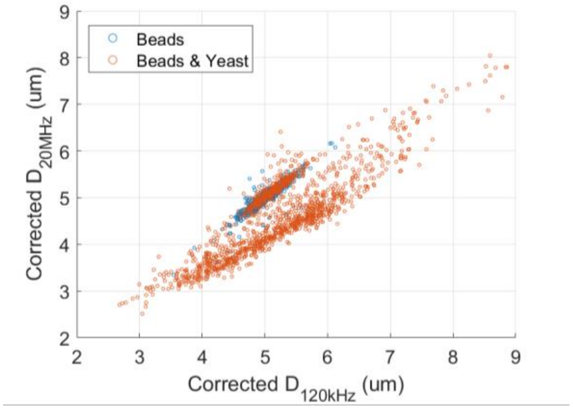

Figure S5: Corrected diameter D at 20MHz and 120 kHz.

Literature overview

Table S1: Comparison of the presented method to other work. The coefficient of variation (CV) of each bead size is defined as the standard deviation of the bead diameter over the nominal bead diameter. The experimental CV is the determined using the standard deviation of a Gaussian fit, whereas the manufacturer CV is specified by the manufacturer.

		**Spencer *et al.* 2016 [2]**		**Ninno *et al.* 2017 [3]**		**Errico *et al.* 2017 [4]**		**Caselli *et al.* 2018 [5]**		**This work**	
		Experimental CV (%)	Manufacturer CV (%)	Experimental CV (%)	Manufacturer CV (%)	Experimental CV (%)	Manufacturer CV (%)	Experimental CV (%)	Manufacturer CV (%)	Experimental CV (%)	Manufacturer CV (%)
**Bead size (µm)**	**5**	1.27	1.8	2.4	2.6	4.1	2.6	3.67	2.6	5.8	3.1
	**6**	0.99	7.5*	0.9	10*	1.8	10*	1.89	10*	4.0	2.8
	**7**	1.24	1.7	1.0	1.7	1.9	1.7	1.72	1.7	2.9	1.5
**Number of electrodes**		10 (planar)		5 (coplanar)		3 (coplanar)		4 (coplanar/ liquid electrodes)		2 (coplanar)	

* These manufacturers' values are most likely a conservative estimate.

[1] J. Cottet, A. Kehren, H. van Lintel, F. Buret, M. Frénéa-Robin, P. Renaud, How to improve the sensitivity of coplanar electrodes and micro channel design in electrical impedance flow cytometry: a study, Microfluid. Nanofluidics. 23 (2019) 1–11. https://doi.org/10.1007/s10404-018-2178-6.

[2] D. Spencer, F. Caselli, P. Bisegna, H. Morgan, High accuracy particle analysis using sheathless microfluidic impedance cytometry, Lab Chip. 16 (2016) 2467–2473. https://doi.org/10.1039/c6lc00339g.

[3] A. De Ninno, V. Errico, F.R. Bertani, L. Businaro, P. Bisegna, F. Caselli, Coplanar electrode microfluidic chip enabling accurate sheathless impedance cytometry, Lab Chip. 17 (2017) 1158–1166. https://doi.org/10.1039/c6lc01516f.

[4] V. Errico, A. De Ninno, F.R. Bertani, L. Businaro, P. Bisegna, F. Caselli, Mitigating positional dependence in coplanar electrode Coulter-type microfluidic devices, Sensors Actuators, B Chem. 247 (2017) 580–586. https://doi.org/10.1016/j.snb.2017.03.035.

[5] F. Caselli, A. De Ninno, R. Reale, L. Businaro, P. Bisegna, A novel wiring scheme for standard chips enabling high-accuracy impedance cytometry, Sensors Actuators, B Chem. 256 (2018) 580–589. https://doi.org/10.1016/j.snb.2017.10.113.
